# The complete chloroplast genome of a medicinal plant *Viscum articulatum* Burm.f. (Loranthaceae)

**DOI:** 10.1080/23802359.2021.1962760

**Published:** 2021-08-16

**Authors:** Yuan Zhang, Chongli Deng, Junwen Yang, Dexin Wang, Guobin Deng

**Affiliations:** a Yunnan Academy of Biodiversity, Southwest Forestry University, Kunming, China; b Forestry Resources Exploitation and Utilization Engineering Research Center for Comprehensive Healthcare of Yunnan Provincial Universities

**Keywords:** Parasitic plant, Loranthaceae, chloroplast genome, phylogenomic

## Abstract

*Viscum articulatum* is usually used as famous ethno-medicinal plant and popular drink in many provinces of China. In this study, the characterization of the complete chloroplast genome of *V. articulatum* was analyzed using the Illumina NovaSeq platform. The whole chloroplast genome sequence of *V. articulatum* is 131,825 including a large single-copy region (LSC, 76,069 bp), a small single-copy region (SSC, 8990 bp), and a pair of repeated regions (IRs, 23,383 bp, each). Further gene annotation in our study revealed the chloroplast genome contains 114 genes, including 36 tRNA genes, 8 rRNA genes and 70 protein-coding genes. A total of 118 simple sequence repeats (SSRs) were identified in the chloroplast genome. Phylogenetic development was analyzed based on *V. articulatum* with other species of Loranthaceae, the phylogenetic tree in our study revealed that *V. articulatum* is a lineage independent of other species in genus *Viscum*.

*Viscum articulatum* Burm.f. 1768, a parasitic herbaceous perennial plant, is commonly hosted in ancient tea trees of Yunnan province, southwest China (Wang et al. 2019). It is also distributed widely in India and Bangladesh (Nag et al. 2020). The extracts of this plant contain a wide variety of flavanols, flavonoids, alkaloids, organic acids, and other high molecular weight compounds (Ni et al. 2013). *V. articulatum* is usually used as famous ethno-medicinal plant for the treatment of hemorrhage, pleurisy, gout, heart disease, arthritis, and hypertension (Li et al. 2008). It is also used as a popular drink in many provinces of China, which has been developed into expensive commercial products for sale by tea companies (Wang et al. 2019). For the above reasons, the pharmacognostical and phytochemical investigation of this plant have been intriguing the interests of researchers (Wang et al. 2019; Nag et al. 2020; Najafi et al. 2010).

In recent years, with the increasing demand for *V. articulatum*, also because this plant cannot be grown artificially yet, people have been digging and harvesting wild resources more immoderately. Moreover, because this plant grows very slowly, the resources of *V. articulatum* are significantly decreasing. Considering the chloroplast DNA-based studies can provide invaluable data for studying genetic history and phylogeny, and can also provide important information in conservation and utilization for this species, in this experiment, we collected *V. articulatum* from Mangjin village, Lancang county of Yunnan province (22°1658′N, 100°0223′E, 1545 m above sea level), China. A voucher specimen (YAB 202007, collector: Yuan Zhang, zygogo@163.com) was deposited at Yunnan Academy of Biodiversity, Southwest Forestry University, Yunnan, China. Then we sequenced, assembled and annotated the accurate chloroplast genome with the method of next-generation sequencing. In this study, we firstly reported a whole mitogenome of *V. articulatum* (GenBank ID is MW092828), the results will provide more useful information for phylogenetic and evolutionary research of *V. articulatum*.

For this study, the total genomic DNA of *V. articulatum* was extracted from fresh stem according to the modified CTAB methods (Doyle and Doyle 1987). A genomic shotgun library with an insertion size of 349 bp was constructed, the libraries were sequenced on Illumina NovaSeq platform at Personalbio Biotech (Shanghai, China). The chloroplast genome was assembled using GetOrganelle software version 1.7.1 (Jin et al. 2020), and the assembled chloroplast genome was annotated through the program PGA (Plastid Genome Annotator) (Qu et al. 2019) with *V. coloratum* chloroplast genome (GenBank accession number: KY679303) as a reference, assisted with manual correction. The raw sequencing reads used in this study have been deposited in SRA (accession number: SRR12816271), and the annotated chloroplast genome sequence has been deposited into the GenBank (accession number is MW092828).

The complete chloroplast genome of *V. articulatum* was 131,825 bp and composed of two IRs of 23,383 bp each, which divide a large single copy (LSC) region of 76,069 bp and a small single copy (SSC) region of 8990 bp, the average GC content was 37.50%, with IR regions (43.72%) higher than that in LSC (34.87%) and SSC regions (27.44%). A total of 118 SSR markers ranging from mononucleotide to pentanucleotide repeated motif were identified in the chloroplast genome of *V. articulatum*. The intron-exon structure analysis indicated that 11 genes have introns, among which *atpF*, *clpP*, *rpl2*, *rpoC1*, *rps16*, *trnA-UGC*, *trnG-UCC*, *trnI-GAU*, *trnK-UUU*, *trnL-UAA* have one intron, while *ycf3* have two introns. The chloroplast genomes encoded 114 unique genes, including 36 tRNA genes, 8 rRNA genes and 70 protein-coding genes.

To determine the phylogenetic relationship of *V. articulatum*, based on complete chloroplast genomes of the other 17 species within the family Loranthaceae, chloroplast genomes were downloaded from NCBI, and we chose *Consolida ajacis* and *Aconitum contortum* as outgroups (Figure 1). All chloroplast genomes were aligned using the program MAFFT v7.471 (Rozewicki et al. 2019), and phylogenetic tree (maximum likelihood) constructed by Iqtree software version 1.6.12 (Minh et al. 2020) with 1000 bootstrap replicates, best-fitted model has been confirmed is TVM + F + R2 by ModelTest-NG (Darriba et al. 2020). The phylogenetic analysis revealed that *V. articulatum* is a lineage independent of other species in genus *Viscum* of our study. The study will provide essential data for future research on the phylogenetic and evolutionary relationship of *V. articulatum* and the family Loranthaceae.

**Figure 1.**
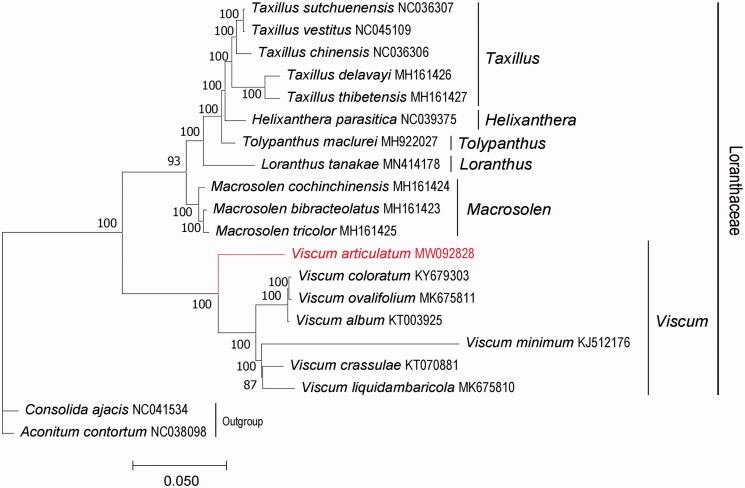
Maximum likelihood (ML) phylogenetic tree based on complete chloroplast genomes of 18 representative species of Loranthaceae and 2 outgroups. Numbers at nodes represent bootstrap values. The genome sequence in this study is highlighted with red text.

## Data Availability

The genome sequence data that support the findings of this study are openly available in GenBank of NCBI at [https://www.ncbi.nlm.nih.gov] (https://www.ncbi.nlm.nih.gov/) under the accession no. MW092828. The associated BioProject, SRA and Bio-Sample numbers are PRJNA668851, SRR12816271, and SAMN16424892 respectively.
